# Linking Motor Competence to Children’s Self-Perceptions: The Mediating Role of Physical Fitness

**DOI:** 10.3390/children12101412

**Published:** 2025-10-20

**Authors:** Ivan Šerbetar, Jan Morten Loftesnes, Asgeir Mamen

**Affiliations:** 1 Department of Kinesiology, Faculty of Teacher Education, University of Zagreb, 10000 Zagreb, Croatia; 2 Department of Education, Art and Sports, Western Norway University of Applied Sciences, 6856 Sogndal, Norway; jan.loftesnes@hvl.no; 3 School of Health Sciences, Kristiania University of Applied Sciences, 0152 Oslo, Norway; asgeir.mamen@kristiania.no

**Keywords:** motor competence, physical fitness, self-perception, mediation, children, MABC-2

## Abstract

**Highlights:**

**What are the main findings?**

- Motor fitness showed small associations with children’s self-perceptions; indirect effects were not supported after false discovery rate control.
- Structural aspects of fitness (strength/size) were unrelated to self-perceptions, and all effects were modest in size.

**What are the implications of the main findings?**

- Functional motor fitness (e.g., agility, balance, endurance) may act as a proximal link between coordination skills and children’s self-concept.
- Physical education that emphasizes diverse motor skills and supportive group contexts may help foster both health and positive self-perceptions.

**Abstract:**

Background/Objectives: Self-perceptions in childhood shape motivation, behavior, and well-being; however, their relationship to motor competence and physical fitness remains unclear. We tested whether physical fitness mediates the association between motor competence and domain-specific self-perceptions in middle childhood. Methods: In a school-based sample of 100 ten-year-olds (59 girls, 41 boys; 3 exclusions ≤ 5th MABC-2 percentile), children completed MABC-2 (motor competence), EUROFIT (physical fitness), and SPPC (self-perceptions). Principal component analysis of the nine EUROFIT tests yielded two factors: Motor Fitness (agility/endurance/flexibility/muscular endurance) and Strength/Size (handgrip and BMI). Parallel mediation models (MABC-2 → [Motor Fitness, Strength/Size] → SPPC) were estimated with maximum likelihood and 5000 bias-corrected bootstrap resamples. Benjamini–Hochberg FDR (q = 0.05) was applied within each path family across the six SPPC domains. Results: In baseline models (no covariates), Motor Fitness → Athletic Competence was significant after FDR (β = 0.263, *p* = 0.003, FDR *p* = 0.018). Associations with Scholastic (β = 0.217, *p* = 0.039, FDR *p* = 0.090) and Social (β = 0.212, *p* = 0.046, FDR *p* = 0.090) were positive but did not meet the FDR threshold. Strength/Size showed no associations with any SPPC domain. Direct effects from MABC-2 to SPPC were non-significant. Indirect effects via Motor Fitness were minor and not supported after FDR (e.g., Athletic: β = 0.067, *p* = 0.106, 95% CI [0.007, 0.174], FDR *p* = 0.251). In BMIz-adjusted sensitivity models, Motor Fitness remained significantly related to Athletic (β = 0.285, *p* = 0.008, FDR *p* = 0.035), Scholastic (β = 0.252, *p* = 0.018, FDR *p* = 0.035), and Social (β = 0.257, *p* = 0.015, FDR *p* = 0.035); MABC-2 → Motor Fitness was β = 0.235, *p* = 0.020. Some paths reached unadjusted significance but were not significant after FDR correction (all FDR *p*-values = 0.120 for indirect effects). Conclusions: Functional Motor Fitness, but not Strength/Size, showed small-to-moderate, domain-specific links with children’s Athletic (and, when adjusting for adiposity, Scholastic/Social) self-perceptions; mediated effects were small and not FDR-supported. Findings highlight the salience of visible, functional performances (e.g., agility/endurance tasks) for children’s self-views and support PE approaches that foster diverse motor skills and motor fitness. Because the study is cross-sectional and BMI-adjusted analyses are presented as robustness checks, caution should be exercised when interpreting the results causally.

## 1. Introduction

### 1.1. Background and Definitions

Self-perception, a multifaceted construct that comprises an individual’s beliefs and feelings about themselves, plays a crucial role in influencing behavior, motivation, and overall well-being, particularly during childhood [1]. Two critical factors of children’s physical development—physical fitness (the capacity to perform physical tasks effectively) and motor competence (the proficiency in executing varied motor skills)—have been recognized as central influences on self-perception [2,3]. The interaction between these physical attributes and self-perception is particularly evident in middle childhood, when children begin to evaluate their competences relative to their peers and incorporate these perceptions into their broader self-concept [4]. In what follows, motor competence refers to observed skill proficiency (MABC-2), physical fitness to performance capacities (EUROFIT), physical activity to behavior (volume/intensity), self-perceptions to domain-specific competence beliefs (SPPC), and adiposity to BMI/BMIz.

Positive self-perceptions are powerful motivators of behavior. Children who view themselves as motor-competent are more likely to engage in and persist with physical activities, reinforcing a virtuous cycle of enhanced fitness and well-being [5]. Conversely, low perceived competence may discourage participation, resulting in inactivity and associated health risks [6]. Indeed, insufficient physical activity in childhood has been associated with a higher risk of chronic diseases such as type II diabetes, hypertension, and impaired mental health outcomes [7,8]. However, global data indicate that most children do not fulfill daily activity guidelines, with the prevalence of inactivity surpassing 80% in some school-aged groups [9,10]. This prevalent shortfall highlights the importance of recognizing motivational and developmental mechanisms that encourage sustained physical activity [11].

### 1.2. Theoretical Framework

The developmental model proposed by Stodden et al. [12] presents one such framework, emphasizing mutually but developmentally sensitive relationships between motor competence, perceived competence, physical activity, and health-related fitness (see also [13,14]). The essence of this model lies in the notion that improvements in motor skill proficiency enhance children’s perceived competence, which in turn encourages increased involvement in physical activity, ultimately leading to improved fitness. This developmental pathway aligns strongly with Harter’s competence motivation theory, which suggests that perceived competence acts as an essential mediator of children’s motivation and involvement in different areas [15]. In relation to the physical domain, this suggests that how children assess their motor abilities can meaningfully influence their willingness to get involved in physical activity, thereby impacting both fitness and broader self-perceptions [16].

### 1.3. Prior Evidence in School-Age Children

Previous research in school-age children provides empirical support for these conceptual models. Cross-sectional studies report that higher motor competence is associated with an increase in perceived competence, higher physical fitness (R2.1), and increased physical activity [17]. Large-scale studies indicate that children with higher perceived motor competence are more active, fitter, and exhibit lower BMI (adiposity), in line with the “spiral of (dis)engagement” [4,12,18]. Longitudinal results also indicate bidirectional relationships between motor skill competence and cardiorespiratory fitness throughout a single school year [19]. Systematic reviews have demonstrated a positive association between motor competence and health-related fitness in youth [20,21] (used here synonymously with “physical fitness”). At the same time, longitudinal evidence suggests that inter-individual differences and developmental changes in motor competence are only partly accounted for by children’s baseline fitness, physical fitness) or adiposity [22], and that children follow heterogeneous growth trajectories in motor competence [23]. Thus, differences in motor-skill development cannot be reduced to “how fit” or “how heavy” children are; rather, motor competence reflects skill-specific proficiencies shaped by practice, instruction, and environmental opportunities [22,23].

### 1.4. Complementary Evidence on Perceived Fitness and Modifiability

Beyond objective testing, children’s self-perceived fitness appears to be a coherent, reliable construct with acceptable concurrent validity against objective tests and related constructs (e.g., IFIS validation in school-age samples and links with physical literacy [24,25,26], complementing findings from self-perception research [27]. Moreover, the accuracy (congruence) between children’s physical self-concept and objectively measured motor competence prospectively predicts higher physical activity one year later, with effects most pronounced among under- or overweight children [28]. Finally, this system is modifiable: a randomized 10-week play-based after-school program improved coordinative abilities (e.g., balance, agility) and physical fitness (e.g., sprint, jump, handgrip) in 12-year-olds [29].

Expanding this framework, the present study examines whether children’s motor competence (assessed by MABC-2) predicts self-perceptions indirectly through their physical fitness (derived from EUROFIT). We treated motor competence as the exogenous predictor, physical fitness as the mediator, and self-perception domains as the outcomes. Notably, while theory often assumes a skill → activity → fitness progression [12,18], recent longitudinal evidence suggests the reverse pathway may not always hold; for instance, a two-year Norwegian follow-up study found that moderate-to-vigorous physical activity predicted later improvements in fundamental motor skills, whereas the reverse path—from motor skills to later physical activity—was not supported [30]. Accordingly, our mediation was defined in a manner consistent with developmental theory and prior evidence, while acknowledging potential reciprocity.

### 1.5. Study Rationale, Gap, and Contribution

Unlike most prior studies that examined isolated pairwise links or used partial fitness batteries, we jointly administered MABC-2, the complete EUROFIT battery (PCA-derived factors), and the SPPC within a single cohort and evaluated a theory-consistent parallel mediation (MABC-2 → fitness → SPPC). To our knowledge, very few studies in middle childhood have implemented this integrative design. In addition, multiplicity control is rarely reported; here we controlled false discoveries within path families (q = 0.05). Recent syntheses suggest that mediating effects are often small or inconsistent, emphasizing the need to test domain specificity and consider adiposity as a potential confounder/modifier—motivating our BMIz-adjusted sensitivity analyses. Guided by developmental models and competence-motivation theory, we specified the ordering motor competence (MABC-2) → physical fitness (EUROFIT) → self-perceptions (SPPC) because motor competence represents foundational skill capacity that enables observable performance capacities (fitness), which in turn shape domain-specific evaluative beliefs (SPPC) in school contexts. Accordingly, we hypothesized that higher motor competence would be associated with more positive self-perceptions indirectly via better physical fitness, such that MABC-2 predicts higher fitness, which in turn predicts higher SPPC scores (positive indirect effect). We further expected domain specificity (stronger effects for athletic competence and physical appearance; minimal for scholastic/social domains).

## 2. Materials and Methods

### 2.1. Participants

We used a school-based convenience cluster sampling approach. Five public elementary schools in Northern Croatia were invited; three participated. Within these schools, complete classes of 10-year-olds were approached during regular physical education lessons to facilitate standardized testing. We focused on 10-year-olds (4th grade) because this point in middle childhood combines sufficient cognitive and social maturity for reliable, domain-specific self-perceptions and peer-referenced comparisons, with consolidating motor competence and measurable fitness. It precedes the curricular transition to subject-specialist PE, creating an ideal opportunity to explore the links among competence, fitness, and self-perceptions. Information sheets and consent forms were distributed to 126 families; 103 children returned parental consent and provided oral assent (response = 81.7%). The inclusion criteria were the absence of reported health or behavioral issues that precluded safe testing. Three children (one boy and two girls) scored at or below the 5th percentile on the MABC-2 and were excluded, in line with our focus on typically developing children, yielding a final analytic sample of 100 (girls, *n* = 59; boys, *n* = 41).

### 2.2. Instruments and Protocol

#### 2.2.1. Self-Perception Profile for Children

The *Self-Perception Profile for Children* (SPPC) [15] is a widely used instrument designed to assess children’s self-concept across multiple domains. The questionnaire consists of 36 items, measuring six subscales: *Scholastic Competence*, *Social Acceptance*, *Athletic Competence*, *Physical Appearance*, *Behavioral Conduct*, *and Global Self-Worth*. Children respond to structured alternative statements, selecting the one that best describes them and then indicating whether the statement is “*really true for me*” or “*sort of true for me*”. This method reduces social desirability bias and promotes genuine self-reflection. Each item is scored on a 4-point scale, with higher scores indicating more positive self-perceptions. The SPPC has demonstrated good reliability and validity across diverse populations and is suitable for children aged 8 to 13 years.

#### 2.2.2. EUROFIT Physical Fitness Test Battery

The EUROFIT (*European Physical Fitness Test Battery*) is a standardized series of physical fitness assessments developed by Adam et al. [31] to evaluate health-related fitness in school-aged children. The battery includes a variety of tests designed to measure different components of physical fitness including: *Flamingo Balance* (static balance), *Plate Tapping* (speed of limb movement), *Sit-and-Reach* (flexibility), *Standing Broad Jump* (explosive leg power), *Handgrip Strength* (muscular strength), *Sit-Ups in 30 s* (abdominal endurance), *10 × 5 m Shuttle Run* (agility), *20 m Shuttle Run (Beep Test)* (cardiorespiratory endurance). In addition, standard anthropometry was obtained (stature and body mass) to compute body mass index (BMI, kg·m^−2^); age- and sex-specific BMI z-scores (BMIz) were derived from reference data and used in sensitivity analyses. The EUROFIT battery has been validated across European populations and is widely used in school and research settings. Standardized protocols ensure consistency in administration and interpretation.

#### 2.2.3. Movement Assessment Battery for Children—Second Edition (MABC-2)

*The Movement Assessment Battery for Children—Second Edition (MABC-2)* [32] is a standardized tool used primarily to identify motor coordination difficulties in children aged 3 to 16 years. Additionally, the MABC-2 is among the most widely used assessments of motor competence globally. This study used *Age Band 2*, which is designed for children aged 7 to 10 years. The battery consists of eight tasks categorized into three domains: *Manual Dexterity* (e.g., *threading lace, drawing a trail*), *Aiming and Catching* (e.g., *catching with one hand*, *throwing at a target*), and *Balance* (e.g., *balancing on one leg*, *walking heel-to-toe)*. Each task is scored according to standardized criteria, with raw scores converted into standard scores and percentile ranks. A total test score below the 5th percentile is indicative of Developmental Coordination Disorder (DCD), while scores between the 6th and 15th percentile suggest a risk of motor difficulties. The MABC-2 is widely recognized for its strong psychometric properties, including test–retest reliability and construct validity [33].

### 2.3. Procedure

Assessments were conducted by four graduate students, who were thoroughly trained in the procedures by the first author. The assessments took place during regular physical education classes. Sessions lasted approximately two school hours per group for the EUROFIT tests and questionnaire completion, while the MABC-2 required about 20 min per participant. Standardized instructions were provided to all participants, and identical equipment and protocols were used across schools to maintain consistency. The assessment phase was completed over a period of approximately two months. Given the restrictive time allocated by schools and tight timetables, and to minimize between-rater variance, we adopted a single-rater-per-station approach: each assessor was responsible for the same test (s) across all children (e.g., one assessor scored all MABC-2 manual dexterity items; another supervised balance). Similarly, EUROFIT stations were run by dedicated assessors throughout the measurement sessions. Children rotated between stations, allowing each specific test to be administered and scored by a designated assessor. Before data collection, the team conducted a brief calibration exercise on an initial subset of participants, in which assessors independently scored the same trials. Discrepancies were discussed, and scoring rules were harmonized. Timed/distance EUROFIT outcomes were obtained with standardized equipment and procedures to reduce scorer subjectivity, and all MABC-2 scoring followed the manual’s operational definitions (e.g., error counts, time criteria).

### 2.4. Data Preparation and Statistical Analysis

Assumptions of normality, linearity, and homogeneity of variance were examined and met before analysis. All EUROFIT test scores were standardized to z-scores to place the variables on a common metric. Standardization was carried out separately for boys and girls to account for well-documented sex differences in absolute performance, ensuring that each child’s score reflected relative performance within their sex group. For tests in which lower values indicate better performance (10 × 5 m Shuttle Run, Plate Tapping, Flamingo Balance), the z-scores were reversed so that higher scores consistently represented better performance across the battery. To describe sex differences, we compared boys and girls on anthropometry, EUROFIT tests, MABC-2 domains, and MABC-2 total using Welch’s *t*-tests (unequal variances and/or sample sizes). We reported Hedges’ g as the standardized mean difference (boys − girls).

Because the EUROFIT battery comprises nine correlated indicators of fitness, separately including all tests in mediation models would have been impractical, given the sample size (N = 103) and the risk of overfitting. To reduce dimensionality and obtain more parsimonious composite measures, a principal component analysis (PCA) was conducted.

The mediation models used MABC-2 (total score) as the predictor, the two PCA-derived EUROFIT factors (Factor 1: Motor Fitness; Factor 2: Strength/Size) as parallel mediators, and each SPPC subscale as the outcome. This ordering follows the developmental rationale outlined in the Introduction. Mediation analyses were conducted using JASP [34]. Models, therefore, tested whether physical fitness components mediated the relationship between motor competence and children’s self-perceptions. Analyses employed maximum likelihood estimation with robust standard errors, and missing data were handled using full-information maximum likelihood. Indirect effects were assessed using 5000 bias-corrected bootstrap resamples, with 95% confidence intervals. As analyses were conducted across six SPPC subscales, a false discovery rate (FDR; Benjamini–Hochberg) correction was examined as a sensitivity check; this did not alter the overall pattern of results.

We report BMIz-adjusted models as sensitivity analyses to examine whether the associations of functional fitness with self-perceptions persist after adjusting for adiposity. Because adiposity may be influenced by motor competence and contributes to Factor 2 (Strength/Size), adjusting for BMIz in mediation can constitute over-control in a strict causal framework. Accordingly, BMIz-adjusted results are presented for robustness rather than as the basis for causal mediation claims.

A priori power was estimated in GPower (v3.1) using a linear multiple regression (R^2^ increase) framework to approximate the mediator → outcome path (controlling for the predictor). For the core models, total predictors were set to k = 3 (MABC-2 predictor and two fitness mediators), with u = 1 tested predictor (the mediator → SPPC path), α = 0.05, and 1 − β = 0.80. Under these settings, a small-to-moderate effect of f^2^ = 0.10 requires approximately N = 85–95; thus our analytic sample (N = 100) is adequate. A sensitivity check indicated that with N = 100 and u = 1, the design can detect effects of about f^2^ = 0.08 (partial R^2^ = 0.07). Because indirect effects were evaluated via bias-corrected bootstrapping (5000 resamples), there is no exact closed-form GPower solution for the mediation test; however, samples around N = 100 typically afford adequate power for small-to-moderate indirect effects when both constituent paths are in the small/medium range. It should be noted that sex was not included as a covariate in the primary mediation models (EUROFIT scores were standardized by sex before PCA); adding a single covariate at this sample size yields a very similar detectable effect size.

### 2.5. Ethics

In accordance with the Declaration of Helsinki, all children and their parents were informed about the purpose and methodology of the study. Parents or legal guardians provided written consent, and children gave oral assent to participate. The study protocol was approved by the Ethics Committee of the Faculty of Teacher Education Zagreb, approval number 2016/11.

## 3. Results

### 3.1. Descriptive Statistics

Descriptive statistics for anthropometric characteristics, fitness tests, and motor competence are presented in Table 1, and self-perception scores in Table 2. In EUROFIT, girls outperformed boys on flexibility (Sit-and-Reach: t = −2.93, df = 85.8, *p* = 0.004, g = −0.592), whereas boys outperformed girls on abdominal endurance (Sit-Ups: t = 2.56, df = 71.5, *p* = 0.013, g = 0.540) and agility (10 × 5 m Shuttle Run: t = −3.67, df = 84.6, *p* < 0.001, g = −0.744). Other EUROFIT tests showed no significant sex differences (all *p* ≥ 0.10). For MABC-2, girls scored higher on Manual Dexterity (t = −3.61, df = 82.7, *p* = 0.001, g = −0.736) and Total score (t = −2.26, df = 64.6, *p* = 0.027, g = −0.488); Aiming & Catching and Balance did not differ significantly by sex (both *p* > 0.30).

Among anthropometric indicators, mean BMI values fell within the normal range; 12 children (11.7%) were classified as overweight and 16 (15.5%) as obese.

Performance on the EUROFIT battery showed some expected sex differences, with boys generally performing better on strength and power measures (Handgrip, Sit-Ups, Standing Broad Jump). In contrast, girls scored higher on flexibility (Sit-and-Reach). Scores on balance (Flamingo), speed of limb movement (Plate Tapping), and agility (10 × 5 m Shuttle Run) were broadly comparable between sexes.

Self-perceptions (SPPC) disclosed a domain-specific profile. Across the sample, the lowest ratings were given on Athletic Competence, suggesting relatively modest self-confidence in sport and movement skills. By contrast, children reported higher self-evaluations for Physical Appearance and Global Self-Worth, indicating generally positive body image and overall self-esteem. Ratings for Scholastic Competence, Social Acceptance, and Behavioral Conduct were moderate and fairly evenly distributed between boys and girls. The internal consistency of the SPPC subscales was acceptable to excellent, with Cronbach’s α values ranging from 0.72 (Social Acceptance) to 0.88 (Global Self-Worth); other scales fell between 0.74 and 0.80.

### 3.2. Principal Component Analysis

A PCA with orthogonal rotation (Varimax with Kaiser normalization) was conducted on the nine EUROFIT test scores to reduce dimensionality and identify latent fitness constructs. Sampling adequacy was verified: the Kaiser–Meyer–Olkin (KMO) measure was 0.74, exceeding the recommended minimum of 0.60, and Bartlett’s test of sphericity was significant (χ^2^ = 288.52, df = 45, *p* < 0.001), confirming that the correlation matrix was factorizable.

The initial analysis yielded three components with eigenvalues greater than one (3.35, 1.81, and 1.10), which together explained 61.4% of the total variance (Table 3). The first two components accounted for 33.5% and 18.1% of the variance, respectively, while the third accounted for 11.0%. Examination of the pattern matrix indicated that the third component was defined almost exclusively by the Bent Arm Hang test, with primary loading of approximately 0.94, and negligible cross-loadings on the other dimensions. In the initial solution, Bent Arm Hang (BAH) formed a single-indicator component (primary loading ≈ 0.94; negligible cross-loadings on all other factors). Our goal was to extract multivariate fitness factors that reflect common variance across multiple tests. Retaining a single-indicator factor would add dimensionality without improving construct coverage or interpretability, give disproportionate weight to one upper-body endurance item, and overlap conceptually with strength/size indicators (e.g., handgrip) without correlating with the broader Motor Fitness profile. We therefore retained the two multifaceted factors (Motor Fitness; Strength/Size).

### 3.3. Mediation Analyses

Parallel mediation models were conducted with the MABC-2 total score as predictor, the two EUROFIT components (Factor 1 and Factor 2) as mediators, and each of the six SPPC subscales as outcomes. Although the MABC-2 provides three domain scores (Manual Dexterity, Aiming & Catching, and Balance), modeling these components separately would have required a complete SEM framework with multiple latent paths, thus necessitating a substantially larger sample size than is available here (N = 100). To maintain parsimony and adequate statistical power, the composite MABC-2 total score was therefore used. All models were estimated with maximum likelihood and 5000 bias-corrected bootstrap samples.

Across outcomes, a consistent pattern emerged: MABC-2 predicted Factor 1 (Motor Fitness); Factor 1 showed small-to-moderate associations with selected self-perception domains; Factor 2 (Strength/Size) was unrelated to SPPC outcomes; and direct effects of MABC-2 on SPPC were uniformly non-significant. In the baseline (no covariates) models, Factor 1 → Athletic Competence was small-to-moderate and remained significant after FDR (β = 0.263; *p* = 0.003; FDR *p* = 0.018), whereas effects for Scholastic (β = 0.217; *p* = 0.039; FDR *p* = 0.090) and Social (β = 0.212; *p* = 0.046; FDR *p* = 0.090) did not meet the FDR threshold. Indirect effects via Factor 1 were positive but small and non-significant (e.g., Athletic: β = 0.067; *p* = 0.106; 95% CI [0.007, 0.174]; FDR *p* = 0.251) and were not statistically supported; indirect effects via Factor 2 were negligible. Where a bootstrap 95% CI touches zero (e.g., Scholastic via Factor 1: −0.000 to 0.165), the indirect effect is imprecise and not distinguishable from zero. In addition, some nominal CIs exclude zero (e.g., Social via Factor 1: 0.001 to 0.157), but the corresponding FDR *p*-value does not meet q = 0.05. Under our multiplicity framework, we treated such effects as exploratory and did not emphasize them. Results for Physical Appearance, Behavioral Conduct, and Global Self-Worth were small and non-significant (e.g., Factor 1 *p* ≥ 0.060; FDR *p* ≥ 0.090).

Baseline (no-covariate) results, including unadjusted and FDR *p*-values, are shown in Table 4. BMIz-adjusted models are summarized next (Table 5), and gender-adjusted models appear in Supplementary Table S1.

Given the potential confounding by adiposity—noting that Factor 2 includes BMI and that adiposity is linked to both fitness performance and self-perceptions—we estimated covariate-adjusted specifications to improve causal interpretability: BMI z-score (BMIz) to account for adiposity, and sex to capture known sex differences in physical performance and self-concept in this age group.

In the BMIz-adjusted models (Table 5), the a-paths indicated MABC-2 → Factor 1 was significant (β = 0.235; *p* = 0.020), whereas MABC-2 → Factor 2 was not (β = 0.057; *p* = 0.324). For the b-paths, Factor 1 predicted Athletic (β = 0.285; *p* = 0.008; FDR *p* = 0.035), Scholastic (β = 0.252; *p* = 0.018; FDR *p* = 0.035), and Social (β = 0.257; *p* = 0.015; FDR *p* = 0.035). Factor 2 → Social was nominally significant (β = −0.415; *p* = 0.024) but did not remain significant after FDR; all other Factor-2 paths were non-significant. Direct effects (c′) from MABC-2 to these outcomes were small and non-significant (Athletic β = −0.065; *p* = 0.531; Scholastic β = 0.110; *p* = 0.287; Social β = −0.089; *p* = 0.385). Indirect effects via Factor 1 or Factor 2 were small; while some were nominally significant before correction (e.g., Athletic via Factor 1: *p* = 0.033; Scholastic via Factor 1: *p* = 0.047), none were statistically supported after FDR (all FDR *p* = 0.120). Full BMIz-adjusted estimates, including unadjusted and FDR *p*-values and bootstrap CIs for indirect effects, are presented in Table 5.

Given the observed sex differences in EUROFIT and MABC-2 (Table 1), we re-estimated the mediation models with sex as a covariate; point estimates were similar and FDR *p* values did not cross 0.05 (see Supplementary Table S1). In those models point estimates for Factor 1 → Athletic/Scholastic/Social were similar in magnitude to the baseline and BMIz-adjusted models (Athletic β = 0.249; *p* = 0.015; Scholastic β = 0.213; *p* = 0.040; Social β = 0.206; *p* = 0.047), but FDR *p*-values were slightly above 0.05 when correcting within the six-domain family (FDR *p* ≈ 0.089–0.094). Factor 2 remained unrelated to SPPC outcomes, with direct effects being non-significant, and indirect effects were not statistically supported after FDR. Detailed results are provided in Supplementary Table S1.

**Figure 1:**
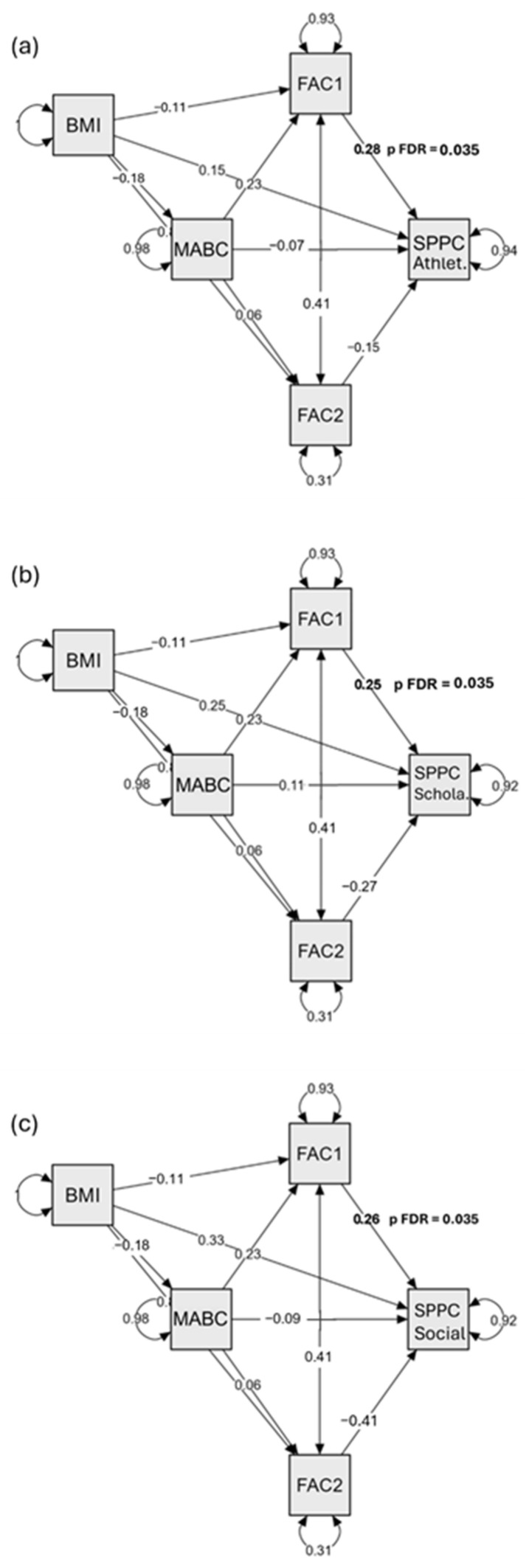
Note. FAC1 = Motor Fitness (agility/endurance/flexibility/muscular endurance; PCA factor); FAC2 = Strength/Size (handgrip, BMI; PCA factor); MABC = MABC-2 total score; SPPC = Self-Perception Profile for Children subscale (Athletic/Scholastic/Social shown in (**a**–**c**)). In BMIz-adjusted models, FAC1 → SPPC was significant after FDR for (**a**) Athletic, (**b**) Scholastic, and (**c**) Social (FDR *p* = 0.035 for each). Direct paths MABC → SPPC were not significant, FAC2 → SPPC paths were not FDR-significant, and indirect (mediated) effects via FAC1 or FAC2 were not supported after FDR. Residual covariance between FAC1 and FAC2 was freely estimated. All coefficients are standardized.

Figure 1a–c BMIz-adjusted mediation models: MABC-2 → (Motor Fitness, Strength/Size) → SPPC domains (Athletic, Scholastic, Social).

## 4. Discussion

### 4.1. Principal Findings and Relative Contribution

This study examined whether physical fitness is associated with the link between motor competence and children’s domain-specific self-perceptions at age 10. Across specifications, Motor Fitness (Factor 1: agility, balance, endurance) was the only consistent correlate of self-perceptions, whereas Strength/Size (Factor 2: grip strength, BMI) showed no associations with any SPPC domain. Direct effects from MABC-2 to SPPC were uniformly non-significant when both fitness factors were included in the model, and indirect (mediated) effects were small and not statistically supported after controlling for the false discovery rate (FDR).

Unlike most prior studies that examined isolated pairwise links or used partial fitness batteries, we jointly assessed motor competence (MABC-2), the complete EUROFIT battery summarized into PCA-derived factors, and domain-specific self-perceptions (SPPC) in a single cohort. We tested a parallel mediation with FDR control and BMIz-adjusted sensitivity—an integrative design that clarifies which domains relate to children’s self-views.

The most evident pattern emerged in the BMIz-adjusted analysis: Motor Fitness was positively related to Athletic Competence, Scholastic Competence, and Social Acceptance after Benjamini–Hochberg FDR correction within the six-domain family (β ≈ 0.25–0.29; FDR *p* = 0.035 for all three). In the baseline (no covariates) models, Motor Fitness showed similar directions and magnitudes—Athletic was significant and Scholastic/Social were positive in unadjusted tests but did not meet the FDR threshold—with only the Athletic path remaining significant after FDR in that analysis. With sex included as a covariate, point estimates were again similar but did not meet the FDR threshold. Taken together, these results indicate small-to-moderate, domain-specific links between functional motor fitness and children’s self-perceptions that are most evident when adiposity (BMIz) is accounted for. Because adiposity may lie on the causal pathway and contributes to Strength/Size, we view the BMIz-adjusted estimates as robustness checks of the associations between Motor Fitness and self-perceptions rather than identified causal mediation effects.

### 4.2. Interpretation and Mechanisms

These results support earlier research indicating that motor competence, perceived competence, fitness, and physical activity cluster in middle childhood [4,17], and there is meta-analytic evidence that physical activity is related to physical self-concept [35]. Our pattern suggests a proximal link between motor competence and self-perception via functional performance capacities rather than structural attributes. This interpretation is consistent with Harter’s competence motivation framework, which states that mastery experiences within a domain shape perceived competence [36], and clarifies why Strength/Size showed no links to any SPPC domain. Similarly to findings by Redondo-Gutiérrez et al. [27], children appear to evaluate themselves more in terms of what they can do (run, jump, balance, finish tasks) than how strong or large they are at this age.

MABC-2 predominantly indexes coordination/skill proficiency under standardized, low-salience conditions, whereas Motor Fitness reflects public, effortful performances that are prominent in everyday PE (e.g., shuttle runs, agility/endurance tasks). In classroom/PE contexts, such visible performance cues likely weigh more heavily than less observable coordination proficiencies when children and peers form Athletic (and to a lesser extent Social) self-perceptions, consistent with evidence that students experience PE as a high-visibility setting in which observable effort and outcomes are salient to classmates [37,38]. At the same time, coordination is linked to academic and cognitive outcomes [39,40], which offers a plausible account of the Scholastic association once multiplicity is controlled through shared planning, sequencing, and executive demands rather than public performance. Notably, the mediated pathways were small and not statistically supported after FDR, indicating that any indirect effect of motor competence on self-perception via fitness appears modest in cross-sectional data. Consistent with Harter’s domain-specific model, these broader facets are shaped primarily by classroom climate, peer acceptance, and teacher feedback, which can overshadow fitness-related cues in this age group [36,41].

### 4.3. Measurement Considerations

The MABC-2 emphasizes manual dexterity, aiming/catching, and balance tasks and does not directly assess locomotor skills (e.g., running, hopping, skipping), which are highly salient in school PE; this measurement focus may partly explain why functional Motor Fitness showed clearer links with self-perceptions [32].

The null role of Strength/Size is informative. Although handgrip, BMI, and Bent-Arm Hang measures structural/muscular capacity, they were not related to self-perceptions in any model, including the BMIz-adjusted models. This supports the view that, near age 10, children’s self-evaluations emphasize functional effectiveness over size/strength, and that broader domains such as Physical Appearance, Behavioral Conduct, and Global Self-Worth are shaped more by psychosocial/contextual influences (peer/teacher feedback, family support) than by fitness per se, in line with Harter’s domain-specific model [36] and school-based evidence on perceived competence and PE climate [41].

### 4.4. Estimation Perspective and Future Work

Finally, the current findings are best read from an estimation-first perspective: effects are small, domain-specific, and reported with multiplicity control. Longitudinal work indicates dynamic, bidirectional relations among motor competence, adiposity, activity, and fitness across development [19,22,23]. Our cross-sectional approach cannot determine the directionality, but it highlights functional fitness as a closer counterpart of children’s Athletic (and to a lesser extent scholastic/social) self-perceptions at age 10, with many other psychosocial factors necessarily contributing (explained variance in SPPC was small, R^2^ ≈ 3–6%). Future studies should test these pathways longitudinally using larger samples, richer measures of classroom/PE context measures, and preregistered multiplicity control. Taken together, this integrative design goes beyond typical pairwise analyses by aligning standardized skill, fitness, and self-perception measures within one framework, which helps explain why functional (Motor Fitness) rather than structural (Strength/Size) attributes map onto children’s domain-specific self-perceptions.

Our pattern of small, domain-specific relations aligns with new syntheses showing that perceived/actual competence pathways yield modest or inconsistent mediation effects in youth and that weight status can influence these relationships. In the studies that are cross-sectional or longitudinal, perceived competence does not serve as a strong mediator between motor competence and activity levels, suggesting that skill and competence beliefs make partly independent contributions to behavior. At the same time, domain-specific cognitive/scholastic links to motor competence continue to arise in longitudinal reviews, consistent with our Scholastic finding once multiplicity is controlled [42,43,44].

Recent research supports the notion that motor competence, perceived competence, health-related fitness, and physical activity are interlinked in school-age youth; however, the effects of mediation are minimal and specific. In late childhood/adolescence, aerobic fitness—not perceived competence—mediated the motor competence-to-physical activity link, with perceived competence showing associations with motor competence and fitness but no mediation of physical activity. This aligns with our finding that functional motor fitness is a better correlate of self-perceptions than structural attributes [43]. Extensive cross-sectional work (N ≈ 2000; ages 4–13) further shows that higher perceived motor competence co-occurs with higher motor competence, fitness, and activity and lower BMI, with patterns that vary by age —consistent with a developmental “spiral” but also indicating that BMI links strengthen later, which fits our BMIz-adjusted robustness pattern [4]. A 2025 structural-equation meta-analysis concludes that perceived physical competence is not a strong mediator of the motor competence → physical activity association (minor absolute and relative indirect effects), supporting our understanding that competence beliefs and performance capacity make partly independent contributions [42].

Regarding the relevance to our domain-specific results, our Athletic/Scholastic/Social associations with the Motor Fitness factor align with newer evidence emphasizing performance capacity as a proximal driver of self-views in PE/school contexts. Two threads are relevant. First, recent pooled and cohort analyses highlight complex, often nonlinear, relationships between motor competence and physical activity, which help explain the modest effects observed at single time points. Second, emerging adolescent data suggest that aerobic fitness is a key pathway linking motor competence to behavior [20]. In parallel, a 2024 meta-analysis links motor competence to executive functions (small–moderate), providing a credible foundation for our Scholastic association via shared cognitive demands (planning, sequencing, cognitive flexibility) [45]. Finally, the abilities we identified as significant can be adjusted. A randomized 10-week, play-based after-school program improved agility, balance, sprinting, jumping, and handgrip in 12-year-olds, highlighting the ability to train coordination and fitness in real-world school settings [29].

Together, these recent findings support the understanding that our effects are small, domain-specific, and close to functional capability—with perceived competence contributing alongside, but not necessarily through, fitness pathways at age 10. This aligns with our null mediation (after FDR) and suggests that longitudinal tests jointly tracking motor competence, fitness, competence beliefs, and BMI will be most informative [43].

### 4.5. Strengths and Limitations

A major strength of this study is the integrated use of three well-validated instruments spanning physical and psychological domains (MABC-2, EUROFIT, and SPPC). This combination allowed for the assessment of developmental pathways across motor skills, fitness, and self-concept. Principal component analysis (PCA) reduced the nine correlated EUROFIT indicators into two interpretable components, thereby enhancing parsimony given the limited sample size. Mediation models with 5000 bootstrap resamples and control of the false discovery rate (FDR) strengthened the transparency of inference. Our sample size met the a priori power target for the tested b-paths; mediation estimates are reported with bias-corrected bootstrap confidence intervals and false discovery rate control.

Cross-sectional design prevents the determination of causal relationships; bidirectional relations among competence, activity, adiposity, and fitness are plausible. This reflects feasibility constraints (single assessment opportunity granted by schools) rather than a preferred theoretical approach. The regional, school-based convenience sampling and class-cluster recruitment were implemented to maintain consistency in testing within PE classes and to adhere to school timetables; however, this limits generalizability beyond the participating schools and region. Measurement coverage is not exhaustive: MABC-2 focuses on manual dexterity, aiming/catching, and balance, and does not directly assess locomotor skills (e.g., running, hopping, skipping), which are very prominent in PE; this instrument choice reflects the study’s focus on standardized coordination assessments but could potentially underestimate locomotor proficiency within motor competence concept. The PCA decision to retain two factors and exclude Bent-Arm Hang was motivated by empirical structure (a third, single-indicator component) and conceptual coherence. While this improves interpretability and model parsimony, it may leave out variance specific to upper-body endurance.

EUROFIT scores were standardized by sex before PCA to reduce known absolute sex differences and to uphold the simplicity of the mediation models; however, this approach results in a trade-off, as sex-related between-group variance in raw performance is not directly modeled in the baseline mediation (this issue is addressed in the gender-adjusted sensitivity models). BMIz was included only in sensitivity analyses. Because adiposity can be influenced by motor competence and contributes to the Strength/Size factor, incorporating BMIz as a covariate may lead to excessive control within a rigid causal framework; we therefore interpret BMIz-adjusted paths as robustness checks, not as recognized causal mediation. Applying BH–FDR multiplicity control reduces Type I error but also lowers power for detecting minor effects in a sample of this size; consequently, indirect effects were minor and not supported after FDR, and several b-paths came very close to the adjusted threshold.

Inter-rater reliability is another constraint. Due to the limited assessment time allocated and tight school schedules, we adopted a single-rater-per-station design rather than repeated ratings on all participants; consequently, formal inter-rater coefficients were not estimated across the full sample.

A brief calibration session at study onset, and most outcomes are objective (time/distance/count). Still, the absence of a full-sample IRR remains a limitation for objectivity and reproducibility. Finally, some contextual covariates (e.g., socioeconomic status, pubertal status, teacher/PE climate) were not collected due to time/privacy constraints; residual confounding is therefore possible.

Together, these considerations suggest a cautious interpretation: effects are small, domain-specific, and most pronounced for functional Motor Fitness. Future longitudinal or intervention designs with preregistered multiplicity control, more covariates (including PA behavior), formal IRR, and expanded locomotor assessment would reinforce causal and ecological claims.

### 4.6. Implications and Future Directions

The results indicate that motor fitness—attributes such as agility, balance, flexibility, and muscular endurance—may serve as a proximal bridge between motor competence and children’s domain-specific self-perceptions. This suggests that school-based physical education should emphasize diverse movement tasks and mastery experiences rather than focusing solely on structural or body-related outcomes. By fostering opportunities to perform motor skills successfully, educators may promote both physical health and more positive self-perceptions in athletic (and, to a lesser extent, scholastic/social) domains. In addition, because functional performances (e.g., running, jumping, or balance tasks) are visible to peers, they may shape not only children’s self-evaluations but also their sense of social acceptance. Encouraging supportive group contexts in which these skills are practiced could therefore enhance both perceived competence and peer relations in physical education settings.

Future research should employ longitudinal or intervention designs to elucidate the sequencing of motor competence, fitness, and self-perceptions across development, and should preregister multiplicity control (e.g., false discovery rate, FDR) given the small effects. Including broader psychosocial variables (e.g., social support, motivational climate, perceived competence from significant others) and contextual covariates (e.g., adiposity, socioeconomic factors) would provide a more comprehensive account of the determinants of self-concept. A recent review highlights that motor competence relates not only to physical and academic outcomes but also to cognitive and socio-emotional domains [44], underscoring the value of integrative, longitudinal approaches. Together, such work can test whether the small, domain-specific associations observed here persist over time and under BMI-informed analytic strategies, placing the present findings in a broader developmental context.

## 5. Conclusions

This study investigated whether physical fitness is linked to motor competence in children’s domain-specific self-perceptions at the age of 10. Across specifications, Motor Fitness (agility, endurance, flexibility, muscular endurance) showed small, domain-specific associations with self-perceptions—most consistently for Athletic Competence in baseline models and, in BMIz-adjusted sensitivity models, also for Scholastic Competence and Social Acceptance after FDR correction. Strength/Size (handgrip, BMI) was unrelated to self-perceptions in any model. Direct paths from motor competence to self-perceptions were not significant, and indirect (mediated) effects via fitness were small and not supported under FDR control.

Taken together, visible, functional performance capacities appear more relevant than structural attributes for children’s self-perceptions at this age. Given the small effect sizes and the cross-sectional design, conclusions should be cautious. Nevertheless, school-based physical education that emphasizes diverse motor skills and functional fitness may support both health and more positive self-perceptions. Longitudinal or intervention studies—with preregistered multiplicity control and broader contextual covariates—are warranted to test these pathways more definitively.

## Figures and Tables

**Table 1:** Anthropometry, EUROFIT, and MABC-2 test scores (Mean ± SD).

Variable	Boys (*n* = 42)	Girls (*n* = 61)	Welch t (df)	*p*	Hedges g (95% CI)
Anthropometry					
Height (cm)	143.84 (7.08)	144.32 (7.03)	−0.339 (87.9)	0.735	−0.068
Weight (kg)	38.50 (9.19)	38.13 (9.15)	0.201 (88.0)	0.841	0.040
BMI	18.66 (3.11)	18.18 (3.40)	0.729 (90.7)	0.468	0.145
Overweight (*n*)	5	4			
Obese (*n*)	7	9			
EUROFIT tests					
Flamingo Balance (errors)	11.15 (6.00)	10.62 (5.40)	0.724 (77.03)	0.471	0.15
Plate Tapping (s)	16.16 (2.21)	16.05 (2.15)	0.218 (84.8)	0.828	0.044
Sit-and-Reach (cm)	17.43 (5.50)	20.71 (5.46)	−2.93 (85.8)	**0.004**	−0.592
Standing Broad Jump (cm)	134.43 (21.67)	133.56 (21.57)	0.811 (85.3)	0.42	0.164
Handgrip Strength (kg)	10.29 (4.06)	8.98 (3.79)	1.63 (82.2)	0.107	0.333
Sit-Ups (30 s)	18.44 (4.57)	16.27 (3.53)	2.556 (71.5)	**0.013**	0.54
Bent Arm Hang (s)	8.50 (5.23)	7.87 (6.70)	0.529 (96.6)	0.598	0.102
Shuttle Run 10 × 5 m (s)	25.32 (2.57)	26.29 (2.35)	−3.671 (84.6)	**<0.001**	−0.744
Shuttle Run 20 m (laps)	27.15 (9.92)	25.78 (8.92)	0.707 (80.1)	0.481	0.146
MABC-2					
Manual dexterity	28.24 (5.53)	32.20 (5.21)	−3.605 (82.7)	**0.001**	−0.736
Aiming & catching	20.32 (5.11)	19.92 (3.58)	0.433 (66.5)	0.666	0.093
Balance	31.90 (4.61)	32.78 (3.37)	−1.043 (68.6)	0.301	−0.222
Total score	80.46 (11.41)	85.11 (7.65)	−2.258 (64.6)	**0.027**	−0.488
≤5th percentile (*n*)	1	2			

Hedges’ g reported (bias-corrected standardized mean difference; boys–girls). Welch’s *t*-tests used (unequal variances). *p*-values shown to 3 decimals; values < 0.001 are reported as *p* < 0.001. Bold *p*-values denote statistical significance (two-tailed α = 0.05).

**Table 2:** Self-perception scores (SPPC; Mean ± SD).

Scale	Boys (*n* = 42)	Girls (*n* = 61)	Welch t (df)	*p*	Hedges g (95% CI)
Scholastic competence	2.94 (0.69)	2.88 (0.69)	2.36 (89.7)	**0.02**	0.471
Social acceptance	2.99 (0.56)	2.87 (0.54)	0.096 (83.2)	0.924	0.02
Athletic competence	2.91 (0.52)	2.65 (0.56)	−0.161 (85.4)	0.872	−0.033
Physical appearance	3.05 (0.69)	3.07 (0.68)	0.441 (86.2)	0.66	0.089
Behavioral conduct	3.03 (0.56)	3.02 (0.53)	0.137 (83.6)	0.891	0.028
Global self-worth	3.13 (0.69)	3.12 (0.66)	1.022 (83.9)	0.31	0.208

Hedges’ g reported (bias-corrected standardized mean difference; boys–girls). Welch’s *t*-tests used (unequal variances). Bold *p*-values denote statistical significance (two-tailed α = 0.05).

**Table 3:** Rotated factor loadings for EUROFIT tests (Pattern Matrix, Varimax rotation).

Test (EUROFIT)	Factor 1 Motor Fitness	Factor 2 Strength/Size	Factor 3
Flamingo Balance	0.26	**−0.67**	0.04
Plate Tapping	**0.61**	−0.01	0.11
Sit-and-Reach	**0.62**	0.19	−0.04
Standing Broad Jump	**0.72**	−0.36	−0.28
Handgrip Strength	0.43	**0.73**	−0.15
Sit-Ups	**0.66**	−0.23	0.13
Bent Arm Hang	0.10	−0.03	**0.94**
Shuttle Run 10 × 5	**0.71**	−0.18	−0.09
Shuttle Run 20 m	**0.85**	0.00	0.28
Body mass index	−0.16	**0.84**	0.11

Note: All loadings are displayed; primary loadings are in bold. Bent Arm Hang loaded strongly (0.94) on a third component in the initial three-factor solution but did not load substantially on the two retained factors and was excluded.

**Table 4:** Summary table for mediation analysis (MABC-2 → Fitness → SPPC).

SPPC Outcome	Direct MABC → SPPC	FAC1 → SPPC	FDR (BH) *p*	FAC2 → SPPC	Indirect via FAC1	FDR (BH) *p*	Indirect via FAC2
Scholastic Competence	β = 0.093, *p* = 0.306	β = 0.217, ***p* = 0.039**	0.090	β = −0.058, *p* = 0.536	β = 0.055, *p* = 0.167, CI [−0.000, 0.165]	0.251	β = 0.005, *p* = 0.690, CI [−0.011, 0.051]
Social Acceptance	β = −0.111, *p* = 0.270	β = 0.212, ***p* = 0.046**	0.090	β = −0.146, *p* = 0.150	β = 0.054, *p* = 0.153, CI [0.001, 0.157]	0.251	β = 0.014, *p* = 0.481, CI [−0.009, 0.077]
Athletic Competence	β = −0.075, *p* = 0.426	β = 0.263, ***p* = 0.003**	**0.018**	β = −0.021, *p* = 0.829	β = 0.067, *p* = 0.106, CI [0.007, 0.174]	0.251	β = 0.002, *p* = 0.880, CI [−0.017, 0.042]
Physical Appearance	β = −0.045, *p* = 0.633	β = 0.033, *p* = 0.741	0.74	β = −0.097, *p* = 0.298	β = 0.008, *p* = 0.770, CI [−0.040, 0.081]	0.770	β = 0.009, *p* = 0.554, CI [−0.008, 0.063]
Behavioral Conduct	β = 0.095, *p* = 0.309	β = 0.142, *p* = 0.137	0.164	β = −0.097, *p* = 0.394	β = 0.036, *p* = 0.228, CI [−0.006, 0.120]	0.274	β = 0.009, *p* = 0.598, CI [−0.010, 0.073]
Global Self-Worth	β = −0.005, *p* = 0.955	β = 0.182, *p* = 0.060	0.090	β = −0.042, *p* = 0.697	β = 0.046, *p* = 0.158, CI [0.002, 0.140]	0.251	β = 0.004, *p* = 0.793, CI [−0.015, 0.054]

Note: Estimates are standardized regression coefficients (β). Indirect effects were evaluated using 5000 bias-corrected bootstrap samples; 95% confidence intervals are reported. Bold values indicate significant unadjusted effects (*p* < 0.05). Multiplicity: FDR (Benjamini–Hochberg) was applied within each path family (q = 0.05). Interpretation: If a 95% bootstrap CI includes or touches zero (e.g., −0.000 to 0.165), the indirect effect is not reliably different from zero. Cases where a nominal CI excludes zero but FDR *p* ≥ q (e.g., Social via Factor 1: CI 0.001–0.157; FDR *p* = 0.251) should be regarded as exploratory. Factor 1 = Motor Fitness; Factor 2 = Strength/Size.

**Table 5:** Summary table for mediation analysis controlling for BMI z-score (MABC-2 → Fitness → SPPC).

SPPC Outcome	Direct MABC → SPPC	FAC1 → SPPC	FDR (BH) *p*	FAC2 → SPPC	Indirect via FAC1	FDR (BH) *p*	Indirect via FAC2
Scholastic competence	β = 0.110, *p* = 0.287	β = 0.252, ***p* = 0.018**	**0.035**	β = −0.267, *p* = 0.145	β = 0.059, ***p* = 0.047**, CI [0.000, 0.131]	0.120	β = −0.015, *p* = 0.413, CI [−0.051, 0.033]
Social Acceptance	β = −0.089, *p* = 0.385	β = 0.257, ***p* = 0.015**	**0.035**	β = −0.415, ***p* = 0.024**	β = 0.060, *p* = 0.087, CI [−0.003, 0.142]	0.120	β = −0.024, *p* = 0.367, CI [−0.061, 0.036]
Athletic competence	β = −0.065, *p* = 0.531	β = 0.285, ***p* = 0.008**	**0.035**	β = −0.147, *p* = 0.423	β = 0.067, ***p* = 0.033**, CI [0.006, 0.163]	0.120	β = −0.008, *p* = 0.693, CI [−0.043, 0.018]
Physical Appearance	β = −0.052, *p* = 0.628	β = 0.018, *p* = 0.866	0.866	β = −0.013, *p* = 0.943	β = 0.004, *p* = 0.833, CI [−0.056, 0.073]	0.833	β = −0.001, *p* = 0.940, CI [−0.026, 0.034]
Behavioral Conduct	β = 0.124, *p* = 0.226	β = 0.203, ***p* = 0.053**	0.063	β = −0.463, ***p* = 0.012**	β = 0.048, *p* = 0.100, CI [−0.012, 0.117]	0.120	β = −0.026, *p* = 0.373, CI [−0.078, 0.031]
Global Self-Worth	β = 0.011, *p* = 0.918	β = 0.215, ***p* = 0.046**	0.063	β = −0.242, *p* = 0.195	β = 0.051, *p* = 0.067, CI [−0.004, 0.145]	0.120	β = −0.014, *p* = 0.460, CI [−0.063, 0.019]

Standardized coefficients (β) shown for b-paths (FAC1/FAC2 → SPPC) and c′ (MABC-2 → SPPC); indirect effects via FAC1/FAC2 estimated with 5000 bias-corrected bootstrap resamples. Benjamini–Hochberg FDR applied within each path family across the six SPPC domains (q = 0.05); both unadjusted and FDR *p*-values are reported. No indirect effects via FAC2 remained significant after FDR correction; all FDR *p*-values were greater than 0.120. Bold *p*-values denote statistical significance (two-tailed α = 0.05).

## Data Availability

Data is unavailable due to privacy and ethical restrictions.

## Supplementary Materials

The following supporting information can be downloaded at: https://www.mdpi.com/article/10.3390/children12101412/s1, Table S1: Summary table for mediation analysis controlling for gender (MABC-2 → Fitness → SPPC).

## Abbreviations

The following abbreviations are used in this manuscript:

MABC-2	Movement Assessment Battery for Children-2
SPPC	Self-Perception Profile for Children
FDR	False discovery rate
