# Supplementary material for: Linking Motor Competence to Children’s Self-Perceptions: The Mediating Role of Physical Fitness
Source: Children (Basel). 2025 Oct 20;12(10):1412. doi: 10.3390/children12101412 (PMC12564408; doi:10.3390/children12101412)
Supplement: Supplementary file 1 [file children-12-01412-s001.zip › children-3899038-supplementary.docx]

Supplementary Table S1: Summary table for mediation analysis controlling for gender (MABC-2 → Fitness → SPPC).

| **SPPC outcome** | **Direct MABC → SPPC** | **FAC1 → SPPC** | FDR (BH) p | **FAC2 → SPPC** | **Indirect via FAC1** | FDR (BH) p | **Indirect via FAC2** |
| --- | --- | --- | --- | --- | --- | --- | --- |
| Scholastic competence | β = 0.111,  p = 0.297 | β = 0.213,  ***p* = 0.040** | 0.094 | β = -0.056,  *p* = 0.573 | β = 0.058, *p* = 0.093, CI [-0.003, 0.131] | 0.170 | β = 0.006, *p* = 0.727, CI [-0.025, 0.035] |
| Social Acceptance | β = −0.090,  p = 0.400 | β = 0.206,  ***p* = 0.047** | 0.094 | β = -0.144,  *p* =0.150 | β = 0.056, ***p* = 0.047**, CI [0.000, 0.141] | 0.140 | β = 0.015, *p* = 0.360, CI [-0.014, 0.058] |
| Athletic competence | β = −0.017,  p = 0.866 | β = 0.249,  ***p* = 0.015** | 0.089 | β = -0.015,  *p* = 0.874 | β = 0.067, ***p* = 0.013**, CI [0.008, 0.157] | 0.080 | β = 0.002, *p* = 0.860, CI [-0.021, 0.037] |
| Physical Appearance | β = −0.052,  p = 0.632 | β = 0.034,  *p* = 0.746 | 0.746 | β = -0.098,  *p* = 0.342 | β = 0.009, *p* = 0.720, CI [-0.049, 0.085] | 0.720 | β = 0.010, *p* = 0.527, CI [-0.015, 0.044] |
| Behavioral Conduct | β = 0.103,  p = 0.338 | β = 0.140,  *p* =0 0.182 | 0.218 | β = -0.097,  *p* = 0.340 | β = 0.038, *p* = 0.180, CI [-0.028, 0.105] | 0.216 | β = 0.010, *p* = 0.627, CI [-0.021, 0.062] |
| Global Self-Worth | β = −0.002,  p = 0.987 | β = 0.181,  *p* = 0.087 | 0.131 | β = -0.042,  *p* = 0.680 | β = 0.049, *p* = 0.113, CI [-0.011, 0.150] | 0.170 | β = 0.004, *p* = 0.787, CI [-0.032, 0.046] |

Bold p-values denote statistical significance (two-tailed α = 0.05).
